# *Curcuma longa* rhizome extract and Curcumin reduce the adhesion of *Acanthamoeba triangularis* trophozoites and cysts in polystyrene plastic surface and contact lens

**DOI:** 10.1016/j.ijpddr.2020.11.001

**Published:** 2020-11-17

**Authors:** Watcharapong Mitsuwan, Suthinee Sangkanu, Chonticha Romyasamit, Chalermpon Kaewjai, Tajudeen O. Jimoh, Maria de Lourdes Pereira, Abolghasem Siyadatpanah, Sunil Kayesth, Muhammad Nawaz, Mohammed Rahmatullah, Mark S. Butler, Polrat Wilairatana, Christophe Wiart, Veeranoot Nissapatorn

**Affiliations:** a School of Allied Health Sciences, Southeast Asia Water Team (SEA Water Team), World Union for Herbal Drug Discovery (WUHeDD), and Research Excellence Center for Innovation and Health Products (RECIHP), Walailak University, Nakhon Si Thammarat, Thailand; b Akkhraratchakumari Veterinary College, Walailak University, Nakhon Si Thammarat, Thailand; c Faculty of Medical Technology, Rangsit University, Pathum Thani, Thailand; d Faculty of Pharmaceutical Sciences, Department of Pharmacognosy and Pharmaceutical Botany, Chulalongkorn University, Bangkok, Thailand; e Department of Biochemistry, Habib Medical School, Islamic University in Uganda, Kampala, Uganda; f CICECO-Aveiro Institute of Materials & Department of Medical Sciences, University of Aveiro, Aveiro, Portugal; g Ferdows School of Paramedical and Health, Birjand University of Medical Sciences, Birjand, Iran; h Department of Zoology, Deshbandhu College, University of Delhi, Delhi, India; i Department of Nano-Medicine Research, Institute for Research and Medical Consultations (IRMC), Imam Abdulrahman Bin Faisal University, Dammam, Saudi Arabia; j Department of Biotechnology & Genetic Engineering, University of Development Alternative Lalmatia, Dhaka, Bangladesh; k Institute for Molecular Bioscience, University of Queensland, Brisbane, Australia; l Department of Clinical Tropical Medicine, Faculty of Tropical Medicine, Mahidol University, Bangkok, Thailand; m School of Pharmacy, University of Nottingham Malaysia Campus, Selangor, Malaysia

**Keywords:** *Acanthamoeba triangularis*, Adhesion, *Curcuma longa* extract, Curcumin, Contact lens, Acanthopodia

## Abstract

*Curcuma longa* and Curcumin have been documented to have a wide spectrum of pharmacological effects, including anti-*Acanthamoeba* activity. Hence, this study sought to explore the anti-adhesion activity of *C. longa* extract and Curcumin against *Acanthamoeba triangularis* trophozoites and cysts in plastic and contact lenses. Our results showed that *C. longa* extract and Curcumin significantly inhibited the adhesion of *A. triangularis* trophozoites and cysts to the plastic surface, as investigated by the crystal violet assay (P < 0.05). Also, an 80–90% decrease in adhesion of trophozoites and cysts to the plastic surface was detected following the treatment with *C. longa* extract and Curcumin at 1/2 × MIC, compared to the control. In the contact lens model, approximately 1 log cells/mL of the trophozoites and cysts was reduced when the cells were treated with Curcumin, when compared to the control. Pre-treatment of the plastic surface with Curcumin at 1/2-MIC reduced 60% and 90% of the adhesion of trophozoites and cysts, respectively. The reduction in 1 Log cells/mL of the adhesion of *A. triangularis* trophozoites was observed when lenses were pre-treated with both the extract and Curcumin. Base on the results obtained from this study, *A. triangularis* trophozoites treated with *C. longa* extract and Curcumin have lost strong acanthopodia, thorn-like projection pseudopodia observed by scanning electron microscope. This study also revealed the therapeutic potentials of *C. longa* extract and Curcumin, as such, have promising anti-adhesive potential that can be used in the management/prevention of *A. triangularis* adhesion to contact lenses.

## Graphical abstract

**Figure:**
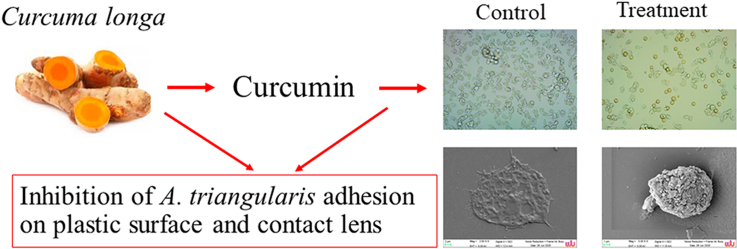

## 1 Introduction

*Acanthamoeba triangularis* (*A. triangularis*) is a free-living protozoan ubiquitously found in nature, such as water and soil. The parasite is a causative agent of several diseases, including granulomatous amoebic encephalitis (Kalra et al., 2020) and *Acanthamoeba* keratitis (Jercic et al., 2019). In keratitis disease, severe vision loss and complete blindness caused by parasites are the main case in contact lens users (Lee et al., 2017). Hence, contact lenses are considered the main risk factor for the transmission *Acanthamoeba* trophozoites and cysts to the eyes (Ibrahim et al., 2009). The adhesion of *Acanthamoeba* to the host cells displays a crucial first step in the pathogenesis of keratitis (Garate et al., 2005). *Acanthamoeba* trophozoites have been reported to show ability to adhere to contact lenses via acanthopodia, thorn-like projection pseudopodia (Lee et al., 2017). Hence, removal of the parasite from the contact lenses is difficult due to the presence of the pseudopodia of the organism. In addition, *Acanthamoeba* cysts resist to antimicrobial substances, resulting in prolonged treatment of the infection.

In an attempt to overcome the infections caused by the parasite, targeting natural products for their anti-*Acanthamoeba* properties could be used for treatment. Phytochemicals isolated from medicinal plants have been focused to treat *Acanthamoeba* infection due to the potent activities of their bioactive molecules (Derda et al., 2016; Dodangeh et al., 2018). Alternative strategies for treating the infection are anti-virulence factors, including anti-adhesion activity (Chen et al., 2013). Strategies are popular in bacterial treatment (Ahmad et al., 2014; Chen et al., 2013). Medicinal plants have been used to treat *Pseudomonas aeruginosa*, a causative agent of contact lens contamination and eye infection (Pratiwi et al., 2014; Zameer et al., 2016). So far, there were a few studies done on *Acanthamoeba* spp.

The present study is focused on the ethanol extract of the rhizome of *Curcuma longa* (*C. longa*), belonging to *Zingiberaceae* family. The plant species has been traditionally used to treat many infections. In addition, it has been documented to have a wide spectrum of pharmacological effects, including anti-oxidant (Tanvir et al., 2017), anti-inflammatory (Lee et al., 2020), and anti-cancer (Ahmad et al., 2020) activities. The rhizome of *C. longa* is the most commonly used part for the medicinal purposes. The rhizome contains several phytochemical compounds, including Curcumin, a yellow pigment in spice turmeric. Curcumin is well known as the main bioactive compound isolated from the rhizome of the plant species. This compound exhibited significant biological activity as antibacterial (Mody et al., 2019) and anti-cancer (Baldi et al., 2020) agents. Furthermore, dimethoxy curcumin, curcuminoids isolated from *C. longa* rhizomes, showed significant amoebicidal effects against *A. castellanii* (Aqeel et al., 2012). Recently, both *C. longa* extract and Curcumin exhibited anti-*Acanthamoeba* activity against *A. triangularis* trophozoites and cysts as published by our research group (Mitsuwan et al., 2020). Having considered the dearth of information on the relevance of natural products as an anti-adhesive agent against the adhesion of parasites, this study is therefore focused on the investigation of the anti-adhesion activity of *C. longa* extract and Curcumin against *A. triangularis* trophozoites and cysts in polystyrene plastic and contact lenses. Polystyrene plastic has been commonly used to determine adhesion activity of many organisms, including *Acanthamoeba* spp. (Imbert-Bouyer et al., 2004). *Acanthamoeba* keratitis in humans is usually transmitted to patients through contact lens users (Ibrahim et al., 2009). In this study, effects of *C. longa* extract and Curcumin on the adhesion of *A. triangularis* on the plastic surface and contact lens was investigated. The activity of *C. longa* extract and the pure compound to prevent the adhesion of *A. triangularis* on both surfaces were further demonstrated. In addition, the morphology of *A. triangularis* trophozoites and cysts after treatment with *C. longa* extract and Curcumin was observed. Preparation of contact lens care multipurpose solutions using *C. longa* extract or Curcumin could be used as an alternative strategy to remove *A. triangularis* trophozoites and cysts in the future.

## 2 Materials and methods

### 2.1 Axenic cultivation of the parasite

*Acanthamoeba triangularis* WU19001 was cultured in Non-nutrient agar plates (NNA plates) seeded with the suspension of *Escherichia coli* (*E. coli*) cells (Mitsuwan et al., 2020). Trophozoite cells were harvested after 48–72 h of incubation at room temperature. Cells were washed twice with Page's saline solution and centrifuged at 4000 rpm for 5 min. The trophozoite viability was investigated using the trypan blue exclusion assay. Trophozoites were adjusted to a final concentration of 2 × 10^5^ trophozoites/mL. The cysts on the NNA plates were harvested after incubating the cultures for 1 week. The preparation and viability of the cysts were performed by the protocol described above.

In order to perform axenic cultivation, Peptone-Yeast Extract-Glucose (PYG) medium (20 g proteose peptone, 18 g glucose, 2 g yeast extract, 1 g sodium citrate dihydrate, 0.98 g MgSO_4_ × 7H_2_O, 0.355 g Na_2_HPO_4_ × 7H_2_O, 0.34 g KH_2_PO_4_, 0.02 g Fe(NH_4_)_2_ (SO_4_)_2_ × 6H_2_O, and 1000 mL distil water) were used as described (Niyyati et al., 2013) with minor modification. The medium was supplemented with 100 unit/mL of penicillin-streptomycin and 10 μg/mL of gentamycin. Briefly, cysts of the parasite were harvested from NNA plates in Page's amoeba saline (PAS) containing the antibiotic. The cysts were cultured in the supplemented PYG to obtain trophozoites, incubated at 25 °C for 48 h. Then, the old medium was removed and replaced with fresh medium every 48 h until 1 month. After that, the trophozoites were cultured in PYG without antibiotic until used. The cysts in PYG were harvested when the cultures were incubated for 1–2 weeks without fresh medium removal. They have fully homogenic inoculum of mature cyst.

### 2.2 Preparation of plant extract and antimicrobial agents

Fifty grams of dried power of *C. longa* rhizome were extracted in 200 mL of 95% ethanol for 7 days. The extracted solution was filtered and evaporated under reduced pressure. Curcumin, a pure compound isolated from *C. longa* rhizome was commercially purchased (Sigma-Aldrich, Missouri, USA). Chlorhexidine included as a positive control, was also purchased (Sigma-Aldrich, Missouri, USA). The extract and the compounds were dissolved in 100% dimethyl sulfoxide (DMSO) and stored at 4 °C.

### 2.3 Effects of media on adhesion of *A. triangularis* to polystyrene plastic surface

In order to investigate the adhesion of *A. triangularis* on the plastic surface, the parasite was cultured in both PYG medium and NNA plates as described above. The adhesion of the trophozoites and cysts of *A. triangularis* were determined as described (Sudjana et al., 2012) with slight modification. *Acanthamoeba* cells from NNA plates were then performed in PYG (NNA-PYG) and PYG supplemented with 100 unit/mL of penicillin-streptomycin (NNA-PYG + antibiotic). Briefly, 100 μL of the parasite inoculum at 3 × 10^5^ cells/mL was added to 96 well polystyrene microtiterplate (culture area = 0.33 cm^2^, recommended working volume = 0.075–0.2 mL, VWR International, USA) containing PYG medium, incubated at room temperature for 24 and 48 h. The old medium was removed to eliminate non-adhesive cells. Then, the wells were washed twice by PAS, and stained by 0.1% crystal violet for 30 min. Plates were washed twice by sterile distil water, and air dried. The stained cells were dissolved in 200 mL DMSO. The inhibitory activity was investigated at optical density 570 nm. The relative percentage of the adhesion was defined as: (mean A570 nm of treated well/mean A570 nm of control well) × 100.

### 2.4 Effects of *C. longa* extracts and curcumin on adhesion of *A. triangularis* to the plastic surface

Effects of *C. longa* extract and Curcumin on adherence of *A. triangularis* trophozoites and cysts were assayed using PYG medium in 96 wells as described (Sudjana et al., 2012). Both trophozoites and cysts cells harvested from PYG were cultured in PYG medium containing sub-minimal inhibitory concentrations (sub-MICs) of the extract and/or Curcumin. The concentrations including sub-MICs of the extract and Curcumin used in this study have been reported by our research team (Mitsuwan et al., 2020). Chlorhexidine and 1% DMSO were included as positive and negative controls, respectively. Plates were incubated at 25 °C for 24 and 48 h. The old medium was removed to eliminate non-adhesive cells. Then, the wells were washed twice by PAS, and stained by 0.1% crystal violet for 30 min. Plates were washed twice by sterile distilled water and air dried. The stained cells were dissolved in 200 mL DMSO. The inhibitory activity was investigated at optical density 570 nm. The relative percentage of the adhesion was defined as: (mean A570 nm of treated well/mean A570 nm of control well) × 100.

### 2.5 Prevention of *Acanthamoeba* adhesion to the plastic plates by *C. longa* extracts and curcumin

The activity of *C. longa* extract and Curcumin to prevent the adhesion of the parasite on the plastic surface was performed in 96 well plates as described (Lee et al., 2017) with modification. Briefly, plastic wells were treated with the extract and the compound at sub-MICs, incubated at 4 °C for 24 h. Chlorhexidine and 1% DMSO were included as positive and negative controls, respectively. The old medium containing the extract and/or the compound was removed and replaced with 100 μL of the fresh medium. One hundred microliter of the parasite inoculum at 3 × 10^5^ cells/mL was added to the wells and incubated at room temperature for 24 h. Prevention of *A. triangularis* adhesion to the plastic surface was investigated by the crystal violet assay as described above.

### 2.6 Effects of *C. longa* extracts and curcumin on adherence of *A. triangularis* to contact lens

The effects of *C. longa* extract and Curcumin to reduce the adhesion of the *A. triangularis* on the three types of commercial soft contact lenses (brands A, B, and C) were performed as described (Lee et al., 2017) with slight modification. Three commercial soft contact lenses consist of brand A (diameter = 13.8 mm, base curve = 8.6 mm, Alcon Laboratories Inc, USA), brand B (diameter = 14.2 mm, base curve = 8.6 mm, Bausch & Lomb Ireland, Ireland), and brand C (diameter = 14.1 mm, base curve = 8.6 mm, Vision Science Co, Korea). Briefly, 500 μL of the parasite inoculum at 3 × 10^5^ cells/mL was added to 24 well plate containing 500 μL of PYG medium containing sub-MICs of the extract and/or Curcumin, incubated at room temperature for 24 h. Chlorhexidine and 1% DMSO were included as positive and negative controls, respectively. The contact lenses were washed in PAS to eliminate non-adhesive cells. The lenses were solved in small tubes containing 500 μL of PAS and mixed. Samples were counted using the trypan blue exclusion assay under inverted microscope (Nikon, Tokyo, Japan).

### 2.7 Prevention of *Acanthamoeba* adhesion to the contact lens by *C. longa* extracts and curcumin

The activity of *C. longa* extract and Curcumin to prevent the adhesion of *A. triangularis* on the contact lenses was investigated as described (Lee et al., 2017) with minor modification. Briefly, the lenses were treated with the extract and the compound at sub-MICs incubated at 4 °C for 24 h. The old medium containing the extract and/or the compound was removed and replaced with 500 μL of the fresh medium. Chlorhexidine and 1% DMSO were included as positive and negative controls, respectively. Five hundred microliter of the parasite inoculum at 3 × 10^5^ cells/mL was added to the wells, incubated at room temperature for 24 h. Prevention of *A. triangularis* adhesion to the plastic surface was investigated by both viable count using trypan blue exclusion assay and the crystal violet assay as described above.

### 2.8 Scanning electron microscopy

The morphology of *A. triangularis* trophozoites and cysts after treatment with the extract and Curcumin were observed by scanning electron microscopy (SEM-Zeiss, Munich, Germany) at Equipment Center, Walailak University as described (Mitsuwan et al., 2020). The parasite cells were treated with different concentrations of the extract and the pure compound in a 24-well plate with a sterile glass coverslip, incubated at room temperature for 24 h. Then, discs were washed three times with PBS. Then, discs were fixed with 2.5% glutaraldehyde in PBS for 24 h. Samples were subsequently washed with PBS. After that, discs were dehydrated in a series of graded ethanol (20–100%), mounted on aluminum stubs and allowed to dry using a critical point dryer. Specimens were then coated with gold particles. Then, the morphology of *Acanthamoeba* cells after treatment was subsequently examined under SEM.

### 2.9 Identification of the bacteria

The identification of bacteria in symbiosis of *A. triangularis* was carried out by a molecular technique. The bacterium was isolated from the well of *A. triangularis* growth in PYG without antibiotic. The microorganism was cultured on Tryptic Soy Agar (TSA), incubated at 37 °C for 24 h. The isolated bacteria were identified by amplification of their 16S rRNA genes using universal primers 27F; 5′-AGAGTTTGATCCTGGCTCAG-3′ and 14927F; 5′- GGTTACCTTGTTACGACTT -3′ as described (Hongoh et al., 2003) with minor modification. Briefly, 3–5 colonies of each bacterial isolated were suspended into 50 μL of TE buffer. Then, cell suspension was heated at 95 °C for 5 min, followed by cooling at 4 °C and centrifuge at 10,000 rpm and 1 min after that, the supernatant was kept for PCR testing. PCR were performed in a total volume of 20 μL PCR reaction consisted of 1X PCR buffer (10 mM Tris-HCl, 50 mM KCl, and pH 8.3), 2.5 mM MgCl_2_, 0.4 mM dNTPs, 1U of *Taq* DNA polymerase, 0.5 μM of 16s rDNA primer pair and 2 μL DNA template. The PCR condition was 5 min at 95 °C, followed by 30 cycles of 30 s at 95 °C, 55 s at 60 °C and 72 s at 70 °C and a final extension of 5 min at 72 °C. The PCR products were held at 4 °C until subjected to agarose gel electrophoresis. Sequencing was conducted using 6 Applied Biosystems 3730xl (Macrogen, Seoul, Korea). Sequences were aligned with NCBI database using BLAST search tool to determined sequence similarity (Han et al., 2014).

### 2.10 Determination of composition of total curcumin in *C. longa* extract

In order to identify composition of total Curcumin in *C. longa* extract, high-performance liquid chromatography (HPLC) was investigated as described (Jayaprakasha et al., 2002). The compounds were isolated and detected using HPLC (Thermo Fisher Scientific, Massachusetts, USA), consisting pump-DIONEX Ultimate 3000 pump (Thermo Fisher Scientific, Massachusetts, USA). Separation of curcuminoids was carried out using C18 Hypersil GOLD™ (5 μm; 4.6 × 250 mm), loaded with silica gels. The mobile phase was acetonitrile and 2% acetic acid. A total of 10 μL of the samples were injected into the column for 15 min of running time. The flow rate was 0.8 mL/min. The total Curcumin presented in the extract was compared with the commercial Curcumin as a standard. The HPLC method was validated as described (Jayaprakasha et al., 2002). The analysis was performed in three independent experiments.

### 2.11 Statistical analysis

The experiments were performed in triplicate. All data were recorded and entered using the statistical package software (SPSS Inc. Chicago, IL, USA). Data were expressed as mean ± SD. Statistical analysis was analyzed by the two-tailed unpaired Student's t-test. In all analyzes, P < 0.05 was considered statistically significant.

## 3 Results

### 3.1 Adhesion of *A. triangularis* on plastic surface in different media

**Fig. 1:**
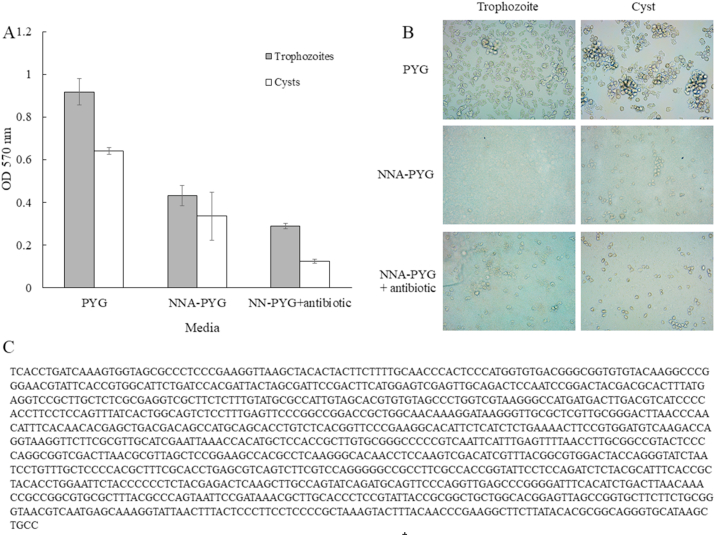
Effects of culture media on the adhesion of *Acanthamoeba triangularis* WU19001 trophozoites and cysts (A). Represents the morphology of the parasite in tested media (B). Gene sequence of 16S rRNA of the bacteria present in NNA-PYG medium was identified as *E. coli* with 99.18% similarity and accession number as MG948940.1 (C).

In order to investigate the adhesion of *A. triangularis* on plastic surface, the organism was cultured in both PYG medium and NNA plates. The adhesion of *A. triangularis* trophozoites or cysts from NNA plates were then assayed in PYG (NNA-PYG) and PYG supplemented with antibiotic (NNA-PYG + antibiotic). Adhesion of both trophozoites and cysts cultured in PYG medium were higher than NNA-PYG and NNA-PYG + antibiotic measured by the crystal violet assay (Fig. 1A). In addition, the density of both trophozoites and cysts in PYG was higher than NNA-PYG and NNA-PYG + antibiotic, as observed by an invested microscope (Fig. 1B). Bacterial growth was observed in adhesion of the parasite in NNA-PYG. The bacterium was isolated and grown on TSA plate. Gene sequence of 16S rRNA of the bacteria presented in NNA-PYG medium was identified as *E. coli* with 99.18% similarity and accession number as MG948940.1 (Fig. 1C). Hence, PYG was chosen as the tested medium for the adhesion experiment.

### 3.2 Inhibition of *A. triangularis* adhesion to plastic surface

**Fig. 2:**
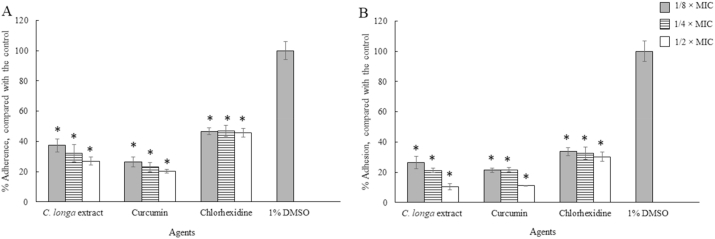
Effects of *Curcuma longa* extracts and Curcumin on adhesion of *Acanthamoeba triangularis* WU19001 trophozoites at 24 h (A) and 48 h (B). The organism was treated with different sub-inhibitory concentrations of the agents, incubated at room temperature for 24 and 48 h. Inhibitory activity was carried out using crystal violet assay. Chlorhexidine and 1% DMSO were used as positive and negative controls, respectively. The relative percentage of the adherence was defined as: (mean of the treated cells/mean of the negative control) × 100, (*significant difference; P < 0.05).

**Fig. 3:**
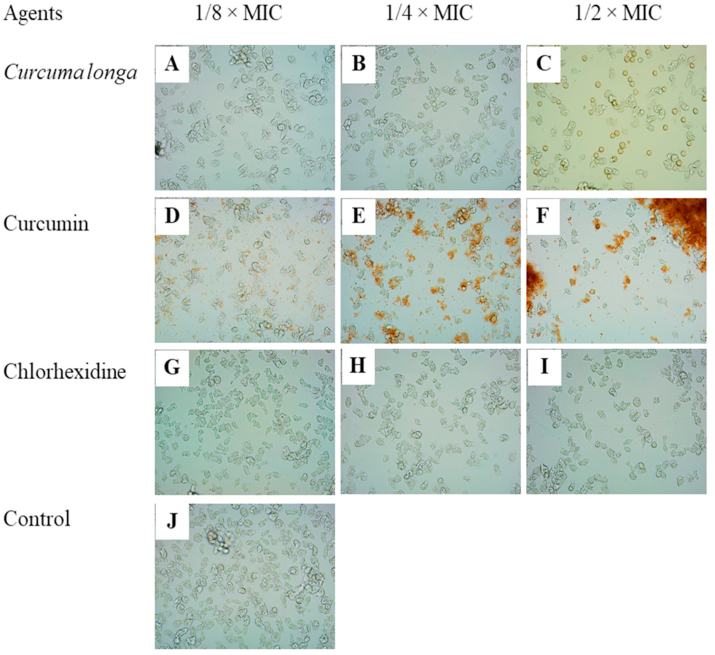
Effects of *Curcuma longa* extract, Curcumin, and chlorhexidine on adhesion of *Acanthamoeba triangularis* WU19001 trophozoites at 24 h. Cells were grown in PYG medium, and treated with the agents at different concentrations, incubated for 24 h. One percent DMSO was included as negative control. Images of the adhesion were observed by inverted microscope (200X).

**Fig. 4:**
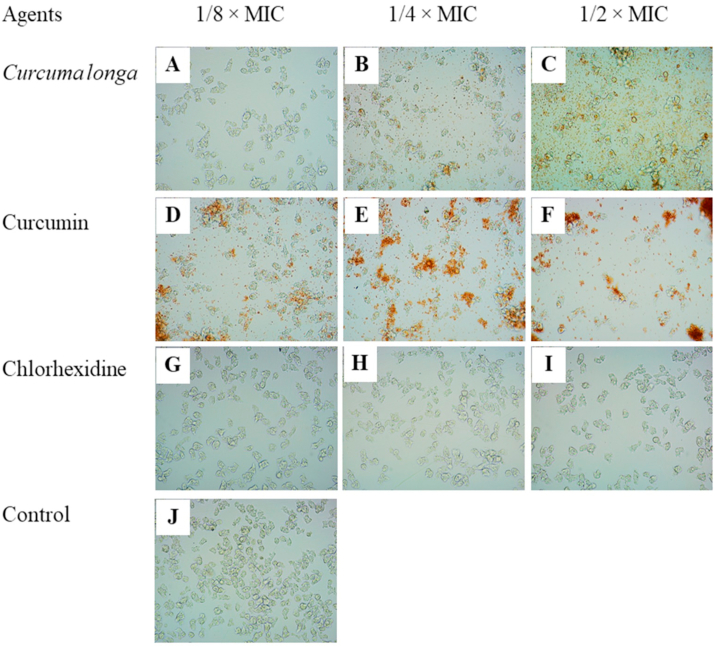
Effects of *Curcuma longa* extract, Curcumin, and chlorhexidine on adhesion of *Acanthamoeba triangularis* WU19001 trophozoites at 48 h. Cells were grown in PYG medium, and treated with the agents at different concentrations, incubated for 48 h. One percent DMSO was included as negative control. Images of the adhesion were observed by inverted microscope (200X).

**Fig. 5:**
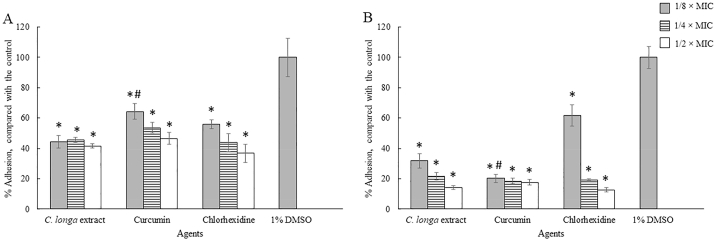
Effects of *Curcuma longa* extracts and Curcumin on adhesion of *Acanthamoeba triangularis* WU19001 cysts at 24 h (A) and 48 h (B). The organism was treated with different sub-inhibitory concentrations of the agents, incubated at room temperature for 24 and 48 h. Inhibitory activity was carried out using crystal violet assay. Chlorhexidine and 1% DMSO were used as positive and negative control, respectively. The relative percentage of the adherence was defined as: (mean of the treated cells/mean of the negative control) × 100, (*, ^#^ significant difference when compared with the control and *C. longa* extract, respectively; P < 0.05).

**Fig. 6:**
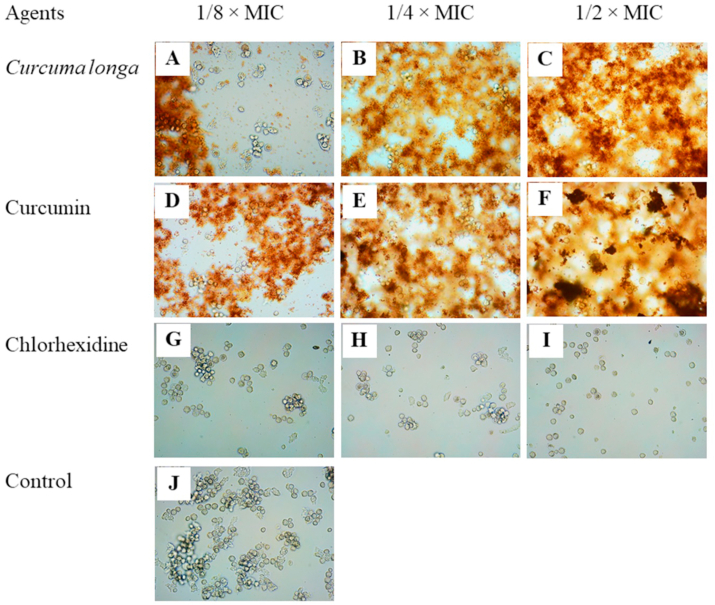
Effects of *Curcuma longa* extract, Curcumin, and chlorhexidine on adhesion of *Acanthamoeba triangularis* WU19001 cysts at 24 h. Cells were grown in PYG medium, and treated with the agents at different concentrations, incubated for 48 h. One percent DMSO was included as negative control. Images of the adhesion were observed by inverted microscope (200X).

**Fig. 7:**
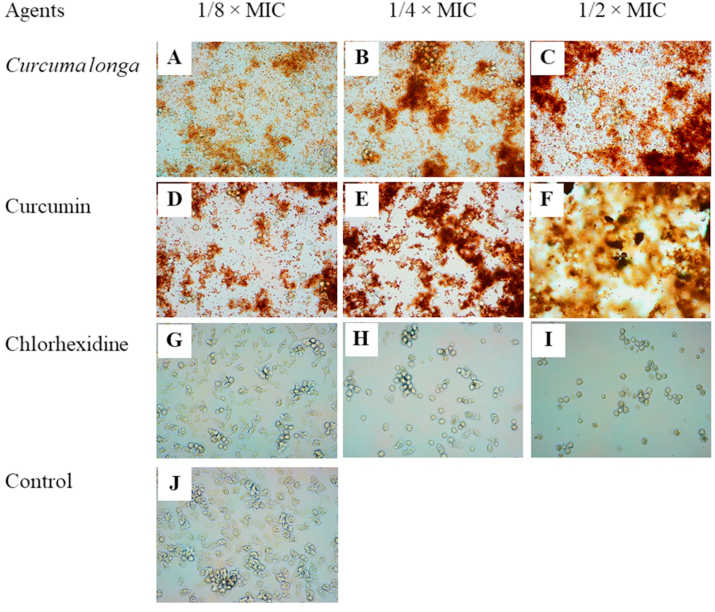
Effects of *Curcuma longa* extract, Curcumin, and chlorhexidine on adhesion of *Acanthamoeba triangularis* WU19001 cysts at 48 h. Cells were grown in PYG medium, and treated with the agents at different concentrations, incubated for 48 h. One percent DMSO was included as negative control. Images of the adhesion were observed by inverted microscope (200X).

In order to investigate the effects of *C. longa* extract and Curcumin on the adhesion of *A. triangularis*, the sub-MICs of both the extract and the compound against the parasite were used (Mitsuwan et al., 2020). The *C. longa* extract and Curcumin significantly inhibited the adhesion of *A. triangularis* trophozoites (Fig. 2, Fig. 3, Fig. 4) and cysts (Fig. 5, Fig. 6, Fig. 7) to the plastic surface (P < 0.05). About 70–80% reduction in *Acanthamoeba* trophozoite adhesion to the surface was observed after 24 h (Fig. 2A), while 90% reduction was also seen in the treated cells at 48 h (Fig. 2B). We however discovered that the triangle cysts of *A. triangularis* were present in *C. longa* treatment at 1/2 × MIC, while the trophozoite form was seen in the control (Fig. 3, Fig. 4).

The inhibition of the adhesion of *A. triangularis* cysts was demonstrated by the treatment with *C. longa* extract and Curcumin, when compared to the control (Fig. 5, Fig. 6, Fig. 7). As shown in Fig. 5B, a fall in 80% adhesion of the cysts to the surface was detected following the treatment with *C. longa* extract and Curcumin at 1/2 × MIC, compared to the control. We also realized that the control cysts have germinated and appeared in trophozoite form, while the treatment groups occurred as cyst form at 48 h (Fig. 7).

### 3.3 Prevention of *A. triangularis* adhesion to plastic surface

**Fig. 8:**
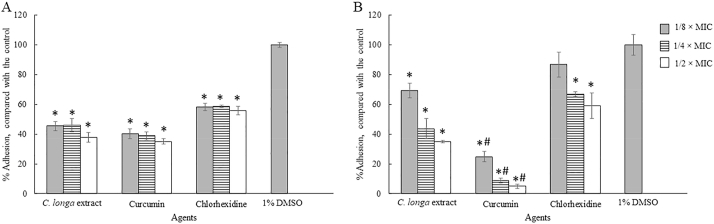
Prevention of the adhesion of *Acanthamoeba triangularis* trophozoites (A) and cysts (B) to the plastic surface by *Curcuma longa* extracts and Curcumin. The surface was treated with the extract and Curcumin at different concentrations, kept at 4 °C for 24 h. Then, the parasitic cells were added, incubated at room temperature for 24 h. Inhibitory activity was carried out using crystal violet assay. Chlorhexidine and 1% DMSO were used as positive and negative controls, respectively. The relative percentage of the adherence was defined as: (mean of the treated cells/mean of the negative control) × 100, (*, ^#^ significant difference when compared with the control and *C. longa* extract, respectively; P < 0.05).

The pre-treatment of the plastic surface with sub-MICs of *C. longa* extract and Curcumin was carried out in polystyrene 96 well plates. Then, surfaces were exposed with the trophozoites and cysts of the organism. The results showed that *C. longa* extract and Curcumin at sub-MICs significantly inhibited *A. triangularis* adhesion to the plastic surface (P < 0.05). As shown in Fig. 8, 60% reduction in the trophozoite adhesion was observed in Curcumin treated groups when compared to the control (Fig. 8A). The pre-treatment of the surface with Curcumin at 1/2-MIC reduced 90% of the cyst adhesion, compared with the control (Fig. 8B). It has been noticed that a significant difference of inhibition of the cyst adhesion was observed following treatment with Curcumin when compared to *C. longa* extract.

### 3.4 Reduction of *A. triangularis* adhesion to contact lens

**Fig. 9:**
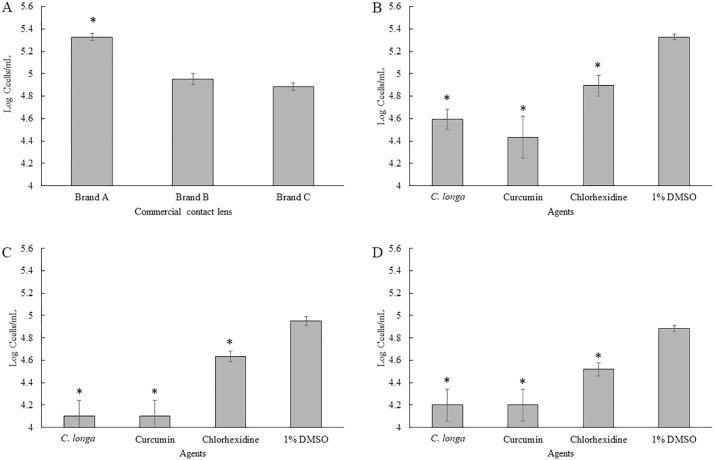
Adhesion of *Acanthamoeba triangularis* WU19001 trophozoites on different commercial contact lens (A). Effects of *Curcuma longa* extracts and Curcumin at 1/2 × MIC on adhesion of A. triangularis trophozoites on the commercial contact lenses including brand A (B), brand B (C), and brand C (D). The data was presented as mean ± SD (*significant difference; P < 0.05).

**Fig. 10:**
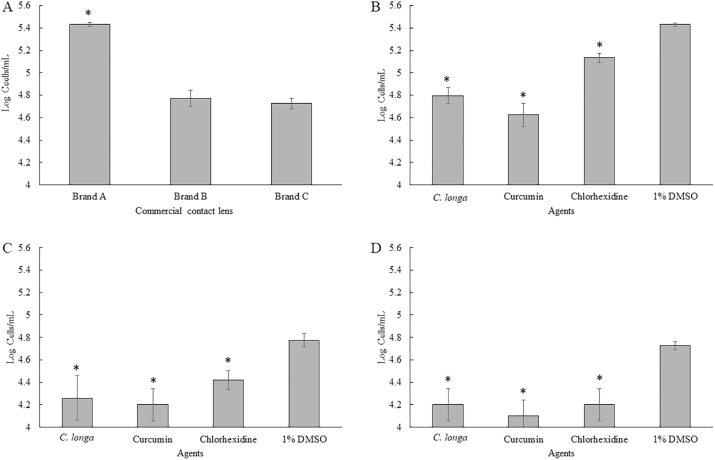
Adhesion of *Acanthamoeba triangularis* WU19001 cysts on commercial contact lens (A). Effects of *Curcuma longa* extracts and Curcumin at 1/2 × MIC on adhesion of A. triangularis cysts on the commercial contact lenses including brand A (B), brand B (C), and brand C (D). The data was presented as mean ± SD (*significant difference; P < 0.05).

In order to apply *C. longa* extract and Curcumin as anti-*Acanthamoeba* agent for cleaning of contact lenses, the activity of *C. longa* extract and the pure compound against *A. triangularis* adhesion to contact lens was investigated. Three commercial contact lens brands were used as contact lens models for the adhesion experiment. Adhesion on contact lens brand A was found to be the most effective when compared with brand B and brand C (Fig. 9A and Fig. 10A). It was observed that the adhesion of the parasite was significantly inhibited by *C. longa* extract and Curcumin at 1/2 × MIC (Fig. 9B–D and 10B-10D). Approximately, 1 log cells/mL of the trophozoites and cysts was lowered when the cells were treated with Curcumin at 1/2 × MIC compared with the control. In addition, *C. longa* extract at 1/2 × MIC slightly inhibited the adhesion of the parasitic cells to the contact lens surface. In addition, significant difference of the inhibition of the adhesion was not observed between *C. longa* extract and Curcumin.

### 3.5 Prevention of *A. triangularis* adhesion to contact lens

**Fig. 11:**
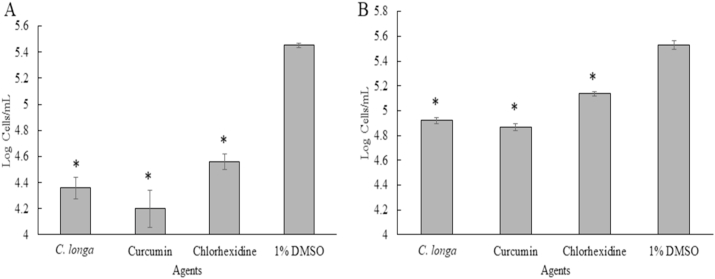
Prevention of the adhesion of *Acanthamoeba triangularis* trophozoites (A) and cysts (B) to the contact lens by *Curcuma longa* extracts and Curcumin. The lenses were treated with the extract and Curcumin at 1/2 × MIC, kept at 4 °C for 24 h. Then, the treated lenses were added with the organism, incubated at room temperature for 24 h. Inhibitory activity was carried out using cell counting by trypan blue exclusion assay. Chlorhexidine and 1% DMSO were used as positive and negative control, respectively. The data was presented as mean ± SD (* significant difference; P < 0.05).

According to the anti-adhesion experiment on contact lenses, adhesion of the parasite on brand A was the highest adhesive, compared with brands B and C. Hence, prevention of *A. triangularis* adhesion to contact lens was carried out using brand A contact lens. In order to apply *C. longa* extract and Curcumin as anti-*Acanthamoeba* agent for cleaning of contact lenses, pre-treatment of the lenses by sub-MICs of *C. longa* extract and Curcumin was carried out. Interestingly, 1 Log cells/mL of the adhesive cells of *A. triangularis* trophozoites was observed when the lenses were treated with both *C. longa* extract and Curcumin (Fig. 11A). However, *C. longa* extract and Curcumin at 1/2 × MIC slightly inhibited the adhesion of the cysts on the contact lens surface (Fig. 11B).

### 3.6 Morphology of *A. triangularis* treated with *C. longa* extract and curcumin

**Fig. 12:**
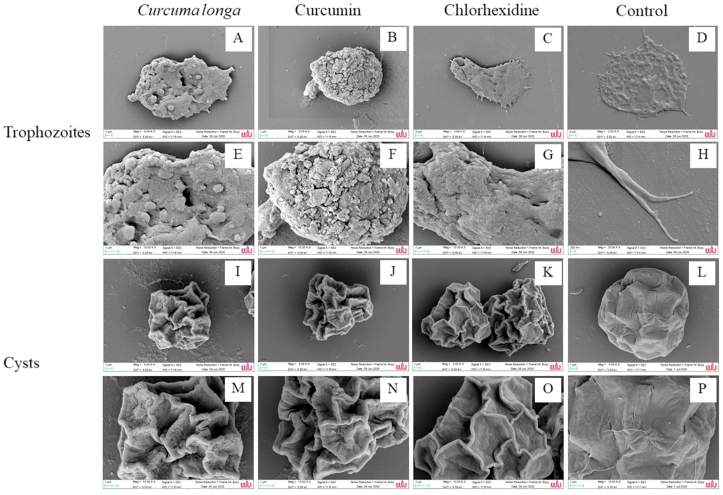
Morphology of *Acanthamoeba triangularis* trophozoites and cysts after treatment with *Curcuma longa* extract and Curcumin. The cells were treated with the extract and Curcumin at 1/2 × MIC. Chlorhexidine and 1% DMSO were used as positive and negative control, respectively. Morphology of the parasite was observed by SEM. Magnifications were revealed as: C, D = 2,500×; A, B, I, J, K, L = 5,000X; E, F, G, M, N, O, P = 10,000X; H = 20,000X

The morphology of *A. triangularis* trophozoites and cysts after treatment with *C. longa* extract and pure Curcumin compound was evaluated by SEM. As shown in the control group, *A. triangularis* trophozoite on the surface demonstrated amoeboid cells with envelope spikes (Fig. 12D). The trophozoite adjacently adhered to the surface using long acanthopodia (Fig. 12D and 12H). The amoeboid trophozoites had lost their ability to each other and started to shrink when the cells were challenged with the agents (Fig. 12A, 12B, 12C, 12E, 12F and 12G). After exposure to *C. longa* extract and Curcumin, trophozoites developed abnormal shape and finally turned to circle cells. It has been highlighted that *A. triangularis* trophozoites treated *C. longa* extract and Curcumin have lost strong acanthopodia. (Fig. 12A, 12B, 12E and 12F). In addition, pore formation was observed when trophozoites were treated with *C. longa* extract (Fig. 12A and 12E). Furthermore, cell membrane of *A. triangularis* was disrupted after interaction with Curcumin (Fig. 12B and 12F).

The morphological cysts with triangle shape and smooth surface were found in the control (Fig. 12L and 12P). It was observed that cysts treated with *C. longa* extract (Fig. 12I and 12M) and Curcumin (Fig. 12J and 12N) at 0.5 × MIC showed forms of retraction when compared to the control. In addition, the cell wall surface of the shrink cysts was slightly disturbed when compared to the control and chlorhexidine treated cells.

### 3.7 Composition of total curcumin in *C. longa* extract

**Fig. 13:**
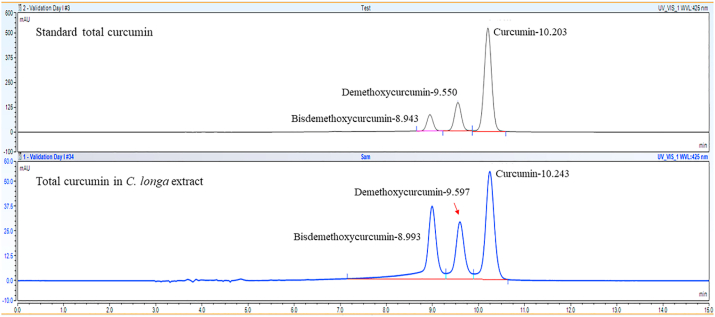
Chromatogram of total Curcumin isolated from *Curcuma longa* extract, compared with standard total Curcumin.

Commercially available Curcumin contains a mixture of three curcuminoids including Curcumin, desmethoxycurcumin, and bisdemethoxycurcumin, as shown by HPLC (Fig. 13). At the same time, composition of total Curcumin in *C. longa* extract was also investigated by HPLC and it was comparatively found that total Curcumin is the major pure compound in the extract.

## 4 Discussion

Free-living amoeba including *A. castellanii*, *A. polyphaga* as well as *A. triangularis* are the leading cases of *Acanthamoeba* keratitis (Jercic et al., 2019). Importantly, severe vision loss and complete blindness caused by these parasites are the main issues in contact lens users (Lee et al., 2017). Therefore, the inhibition of parasite contamination on the contact lenses can be an alternative strategy to reduce the risk of infection. The present study showed the anti-adhesion activity of *C. longa* extract and Curcumin against *A. triangularis* trophozoites and cysts. Also, the axenic culture of *A. triangularis* showed pure parasite cells without bacterial contamination. Essentially, this assay included penicillin-streptomycin and gentamicin to kill the contaminated bacteria (Schuster 2002). Following the best acknowledged culture system, antibiotic was removed from the parasite culture when the bacteria were completely inhibited. Thereafter, parasite cells were cultured in PYG alone throughout the experiment to avoid drug-compound interaction. This critical step however revealed that the cultivated parasite in PYG displayed highest adhesion of *A. triangularis* on the plastic surface when compared with our media used. However, it could be convincingly that culturing of parasite on NNA covered with *E. coli* was easy to perform while bacterial contamination from the plate was observed when cells were grown in PYG. On the other hand, cultivation of the parasite in PYG medium is easy to harvest for the experiment. Therefore, pseudopodia of the organism were not disrupted when compared to culturing on NNA medium. Importantly, pure *Acanthamoeba* cells in PYG medium could be used to study the expression of mRNA, metabolites and proteins without the bacterial genome contamination.

More so, since the presence of glucose in PYG enhances the bacterial overgrowth (Schuster 2002), then the bacteria may rapidly adhere to the surface instead of the parasite. As such, we found the gene sequence of the bacteria in NNA-PYG medium as *E. coli* with 99.18% similarity. Hence, the bacteria could not form symbiosis with *Acanthamoeba* spp. but such relationship could have been established as the bacteria covered on NNA plate as *E. coli*.

In the adhesion of trophozoites to the plastic surface, results have shown that some triangle cysts of *A. triangularis* were present in *C. longa* treatment at 1/2 × MIC, while the trophozoite form was observed in the control. In general, the cyst stage of *Acanthamoeba* spp. occurred in response to various environmental stressors, such as starvation, temperature, pH, osmolality, irradiation, and drugs (Coulon et al., 2010). This study has clearly revealed the role of stress management with emphasis on environmental factors. More importantly, our findings have revealed that the control cysts germinated in trophozoite form while the treatment groups occurred in cyst form at 48 h. This result is in agreement with the previous study of (Niyyati et al., 2013), where experiment was performed in PYG medium suitable for the growth of *Acanthamoeba* and as such, the trophozoites were found within the control system.

Curcumin is the major bioactive compound present in *C. longa* rhizome responsible for various therapeutic and preventive purposes. In our study, we have revealed that *C. longa* extract and its pure compound, Curcumin, exhibited anti-adhesion activity against both trophozoites and cysts of *A. triangularis*. Essentially, our result is in strict conformity with the work of (Alalwan et al., 2017) where Curcumin was reported to have inhibited adhesion and biofilm formation in *Candida albicans* via down regulation of the key adhesins including ALS1 and ALS3. Similarly, Curcumin suppressed quorum sensing activity in urinary tract infected pathogens resulted in biofilm inhibition was reported by (Packiavathy et al., 2014). That the first step in biofilm formation is adhesion of microorganism to the surface is no longer a subject of argument, our results however revealed that individual *A. triangularis* cells adhered to the surface unlike the biofilm.

We have demonstrated that flat and adjacent trophozoites adhered to the surface while the treated trophozoites exhibited shrunken cells. Also, pore formation was observed when trophozoites were treated with *C. longa* extract. Essentially, we have highlighted that trophozoites treated with Curcumin showed the lump shape like cystic form. Moreover, cell surface damage of trophozoites was observed when trophozoites were exposed to Curcumin. As such, we realized that *A. triangularis* trophozoites treated *C. longa* extract and Curcumin have lost strong acanthopodia as revealed by SEM. This result accedes with the study of (Lee et al., 2017) that established a shrunk like cystic shape of *A. lugdunensis* L3a trophozoites, when cells were treated with contact lenses care for multipurpose solutions. In this regard, we resolved that the presence of the shrunken cells after treatment could be a major factor in reducing the surface area of the pathogen to adhere to the plastic and contact lens surfaces.

Acanthopodia thorn-like projection pseudopodia are produced and protruded from every area of the cell's surface. Also, *Acanthamoeba* trophozoites have been reported for their adhesion ability to the contact lens via acanthopodia (Lee et al., 2017). It has been documented that pathogenic *Acanthamoeba* possessed higher number of acanthopodia when compared with non-pathogenic *Acanthamoeba* (Siddiqui et al., 2012). Also, report from (Khan 2001) confirmed that the binding of pathogenic *Acanthamoeba* cells to corneal epithelial cells was mediated by acanthopodia. More importantly, another study from (Khan 2004) submitted that *Acanthamoeba* cells lack acanthopodia and as a result could not bind to corneal epithelial cells. In this regard, it is quintessential to state that, *Acanthamoeba* spp. express a mannose-binding protein that is involved in the adhesion of the parasite to the host cells (Garate et al., 2005) with emphasis on the location of this protein within the parasite's acanthopodia (Khan 2004). Since it has been reported that the down-regulation of mannose-binding protein resulted in reducing *Acanthamoeba* binding to the corneal cells (Garate et al., 2006), then it is not a mere coincidence that *A. triangularis* trophozoites treated *C. longa* extract and Curcumin in this study displayed a strong acanthopodia loss as revealed by SEM. As such, the interference of acanthopodia by *C. longa* extract and Curcumin may inhibit the parasite adhesion. This finding is in line with a previous study showed that acanthopodia of *A. lugdunensis* L3a trophozoites disappeared when cells were treated with contact lens care multipurpose solutions (Lee et al., 2017). In addition, cosmetic contact lenses care multipurpose solution inhibited the adhesion of *A. lugdunensis* to contact lens (Lee et al., 2017). Therefore, the loss of acanthopodia observed in this study may offer a promising therapeutic strategy through which *C. longa* extract and Curcumin could be alternatively used in reducing *A. triangularis* adhesion to the contact lens surface. In addition, Curcumin has been considered as a promising therapeutic candidate for anterior segment eye diseases, including corneal neovascularization, glaucoma, and cataracts that often related to inflammation (Liu et al., 2017). It has been reported that Curcumin could inhibit both TNF-a and IL-1b induced subcellular localization of occludens-1 through NF-kB inhibition (Kimura 2010). This fact may suggest that Curcumin may prevent corneal epithelial barrier function disruption related to the ocular inflammation (Liu et al., 2017).

It is well known that the rhizome of *C. longa* extract contains Curcumin as the main bioactive curcuminoids. However, the composition of the plant material strongly depends on the collected time of the plant and the extraction method. The present study has confirmed that Curcumin was the main bioactive curcuminoids presented in the used *C. longa* extract. In addition, it has been reported that bisdemethoxycurcumin, as one of the minor pure compounds, significantly inhibited the adhesion and invasion of SKOV-3 cells (Pei et al., 2016). While, desmethoxycurcumin also found to inhibit the binding of *A. castellanii* to human brain microvascular endothelial cells (Aqeel et al., 2012). It is therefore suggested that preparation of contact lens care multipurpose solutions using *C. longa* extract or Curcumin could be used as an alternative strategy to remove *A. triangularis* trophozoites and cysts. In addition, the anti-adhesion activity of *C. longa* extract, Curcumin, and contact lens care multipurpose solutions against the parasite could be carried out in other surfaces, including plastic contact lens case. Furthermore, synergistic effects of *C. longa* extract and its compound in combination with antibiotic could be determined as feasible therapeutic agents to combat the infections with *A. triangularis* in the future.

## 5 Conclusion

This study has revealed that *C. longa* extract and Curcumin significantly inhibited the adhesion of *A. triangularis* trophozoites and cysts to plastic surface and contact lenses. Curcumin at 1/2 × MIC reduced 80–90% and 1 log cells/mL adhesion of the trophozoites and cysts to the plastic surface and contact lens when compared with the control, respectively. Interestingly, 1 log cells/mL of the adhesive cells of *A. triangularis* trophozoites was observed when lenses were pre-treated with both *C. longa* extract and Curcumin. It has been highlighted that *A. triangularis* trophozoites treated *C. longa* extract and Curcumin have lost strong acanthopodia. To this end, this study has provided empirical evidence in support of the therapeutic potential of *C. longa* extract and Curcumin as an anti-adhesive agent to prevent *A. triangularis* adhesion to contact lenses.

## Declaration of competing interest

The authors declare no conflict of interest.
